# Commentary: Evaluation of AI-enhanced tele-ECG response time and diagnosis in acute chest pain patients

**DOI:** 10.3389/fcvm.2026.1753595

**Published:** 2026-02-02

**Authors:** Yingying Diao

**Affiliations:** Department of Cardiovascular Medicine, Wenshang County People’s Hospital, Jining, China

**Keywords:** acute chest pain, AI-assisted diagnosis, clinical integration, methodological evaluation, tele-ECG

## 1 Introduction

We read with great interest the article by Accorsi et al. titled “Evaluation of AI-enhanced tele-ECG response time and diagnosis in acute chest pain patients,” recently published in Frontiers in Cardiovascular Medicine (2025) (1). The authors present a valuable real-world analysis of an AI-assisted tele-electrocardiography (tele-ECG) system in emergency settings, highlighting its potential to improve response times and diagnostic efficiency. While the study offers promising insights into the application of artificial intelligence in telemedicine, several methodological aspects warrant further clarification to strengthen the validity and generalizability of the findings.

## 2 Lack of a control group or comparative analysis

A primary concern is the absence of a control group or comparative arm without AI support. Although the authors note that the observed response times are shorter than those reported in previous studies, the lack of a direct within-study comparison limits causal inference regarding the AI's specific contribution. A randomized or matched design comparing AI-assisted versus conventional tele-ECG interpretation would provide more robust evidence of the AI system's incremental benefit, as demonstrated in recent trials such as the ARISE study (2).

## 3 Limited characterization of the AI model

While the study briefly describes the convolutional neural network (CNN) architecture and its internal validation metrics, key details regarding the model's training dataset, external validation, and performance across different demographic or clinical subgroups are not provided. Ensuring transparency throughout the development of AI models by detailing data provenance, labeling methodologies, and inherent biases serves as a cornerstone for achieving reproducibility and building essential trust in clinical settings (3, 4).

## 4 Incomplete reporting of ECG interpretation workflow

The study does not specify whether the same cardiologists interpreted ECGs both with and without AI support, nor does it detail how the AI output was integrated into the final report. To fully appreciate the system's operational role and limitations, it is necessary to clarify the human-AI interaction process. This entails defining whether the AI acted merely as a prioritization tool or also played a part in shaping the diagnostic decisions themselves, a consideration of paramount importance in light of the established inter-rater variability in ECG interpretation (5).

## 5 Underrepresentation of clinical context

The analysis focuses exclusively on ECG tracings and response times, with limited integration of clinical data such as patient symptoms, risk factors, or outcomes. This restricts the ability to assess the AI's impact on clinical decision-making or patient-oriented endpoints (for example, mortality, revascularization success). Future studies should aim to link ECG findings with longitudinal outcomes to evaluate the AI's prognostic utility, as emphasized in recent tele-ECG meta-analyses (6).

## 6 Variability in ECG quality and exclusions

The authors appropriately excluded 12.58% of tracings due to artifacts or technical issues. However, the impact of these exclusions on the overall diagnostic accuracy and workflow efficiency is not discussed. A sensitivity analysis including borderline or suboptimal tracings could provide insight into the system's robustness in real-world conditions, especially given known challenges in pre-hospital ECG transmission (7).

## 7 Generalizability and implementation context

The study was conducted within a well-structured telemedicine network in Brazil. The applicability of these findings to settings with less infrastructure or different patient populations remains unclear. Reporting on barriers to implementation, cost-effectiveness, and scalability would enhance the translational value of the research, particularly for low-resource regions where tele-ECG is most needed (8, 9).

In conclusion, Accorsi et al. have made a noteworthy contribution to the growing body of evidence supporting AI-enhanced telemedicine. Their findings suggest that AI can expedite ECG interpretation and support diagnostic workflows in resource-limited settings. However, to fully establish the clinical utility and reliability of such systems, future studies should incorporate controlled comparisons, detailed model reporting, and broader clinical validation. We commend the authors for their work and hope these considerations will inform subsequent research in this rapidly evolving field.
